# A new glucocerebrosidase-deficient neuronal cell model provides a tool to probe pathophysiology and therapeutics for Gaucher disease

**DOI:** 10.1242/dmm.024588

**Published:** 2016-07-01

**Authors:** Wendy Westbroek, Matthew Nguyen, Marina Siebert, Taylor Lindstrom, Robert A. Burnett, Elma Aflaki, Olive Jung, Rafael Tamargo, Jorge L. Rodriguez-Gil, Walter Acosta, An Hendrix, Bahafta Behre, Nahid Tayebi, Hideji Fujiwara, Rohini Sidhu, Benoit Renvoise, Edward I. Ginns, Amalia Dutra, Evgenia Pak, Carole Cramer, Daniel S. Ory, William J. Pavan, Ellen Sidransky

**Affiliations:** 1 Section on Molecular Neurogenetics, Medical Genetics Branch, National Human Genome Research Institute, National Institutes of Health, Bethesda, MD 20892, USA; 2 Postgraduate Program in Cellular and Molecular Biology, Universidade Federal do Rio Grande do Sul, Porto Alegre, RS 91501-970, Brazil; 3 Genomics, Development, and Disease Section, Genetic Disease Research Branch, National Human Genome Research Institute, National Institutes of Health, Bethesda, MD 20892, USA; 4 Biostrategies LC, State University, AR 72467, USA; 5 Laboratory of Experimental Cancer Research, Department of Radiation Oncology and Experimental Cancer Research, Ghent University Hospital, Ghent 9000, Belgium; 6 Washington University School of Medicine, St. Louis, MO 63110-1093, USA; 7 Cell Biology Section, Neurogenetics Branch, National Institute of Neurological Disorders and Stroke, National Institutes of Health, Bethesda, MD 20892, USA; 8 Lysosomal Disorders Treatment and Research Program, Clinical Labs, University of Massachusetts Medical School, Worcester, MA 01655, USA; 9 Cytogenetics Core, National Human Genome Research Institute, National Institutes of Health, Bethesda, MD 20892, USA

**Keywords:** Gaucher disease, Glucocerebrosidase, Neuron, Glucosylceramide, Glucosylsphingosine

## Abstract

Glucocerebrosidase is a lysosomal hydrolase involved in the breakdown of glucosylceramide. Gaucher disease, a recessive lysosomal storage disorder, is caused by mutations in the gene *GBA1*. Dysfunctional glucocerebrosidase leads to accumulation of glucosylceramide and glycosylsphingosine in various cell types and organs. Mutations in *GBA1* are also a common genetic risk factor for Parkinson disease and related synucleinopathies. In recent years, research on the pathophysiology of Gaucher disease, the molecular link between Gaucher and Parkinson disease, and novel therapeutics, have accelerated the need for relevant cell models with *GBA1* mutations. Although induced pluripotent stem cells, primary rodent neurons, and transfected neuroblastoma cell lines have been used to study the effect of glucocerebrosidase deficiency on neuronal function, these models have limitations because of challenges in culturing and propagating the cells, low yield, and the introduction of exogenous mutant *GBA1*. To address some of these difficulties, we established a high yield, easy-to-culture mouse neuronal cell model with nearly complete glucocerebrosidase deficiency representative of Gaucher disease. We successfully immortalized cortical neurons from embryonic null allele *gba^−/−^* mice and the control littermate (*gba^+/+^*) by infecting differentiated primary cortical neurons in culture with an EF1α-SV40T lentivirus. Immortalized *gba^−/−^* neurons lack glucocerebrosidase protein and enzyme activity, and exhibit a dramatic increase in glucosylceramide and glucosylsphingosine accumulation, enlarged lysosomes, and an impaired ATP-dependent calcium-influx response; these phenotypical characteristics were absent in *gba^+/+^* neurons. This null allele *gba^−/−^* mouse neuronal model provides a much-needed tool to study the pathophysiology of Gaucher disease and to evaluate new therapies.

## Abstract (precis)

**Summary:** This work describes the generation of a novel immortalized glucocerebrosidase-deficient neuronal cell model with utility for pathophysiology research and therapeutic development in Gaucher disease.

## INTRODUCTION

The enzyme glucocerebrosidase (GCase), a lysosomal-resident hydrolase encoded by the gene *glucocerebrosidase* (*GBA1*), is involved in the breakdown of two substrates, glucosylceramide (GlcCer) and glucosylsphingosine (GlcSph). Gaucher disease (GD, MIM #606463), an autosomal recessive lysosomal storage disorder, is caused by mutations in *GBA1*. Deficient GCase leads to lysosomal substrate accumulation in cells of the macrophage lineage and clinical manifestations including organomegaly, anemia, thrombocytopenia, osteopenia and inflammation (Beutler and Grabowski, 2001; Sidransky, 2004). GD is classified into three types: a non-neuronopathic type 1, an acute neuronopathic type 2, and a chronic neuronopathic type 3, with a broad continuum of clinical manifestations in between (Sidransky, 2004). Mutations in *GBA1* have now been established as an important risk factor for the development of synucleinopathies including Parkinson disease (PD) (Sidransky et al., 2009), dementia with Lewy bodies (DLB) (Nalls et al., 2013), and multiple system atrophy (MSA) (Mitsui et al., 2015). Furthermore, GCase enzyme activity and protein expression levels are reduced in select brain regions of individuals with PD without *GBA1* mutations (Murphy et al., 2014; Gegg et al., 2012). Until recently, uncovering GD-associated cellular impairments was challenging because of the lack of relevant cell models. Primary dermal fibroblast cultures established from skin biopsies taken from individuals with GD were the only available cell model to study the biological implications of GCase deficiency, but these cells do not store lysosomal substrate. In recent years, intense research on the link between *GBA1* mutations and synucleinopathies, as well as the development of novel therapeutics, has prompted the development of novel cell models. The majority of neuronal cell models commonly used for such studies include wild-type neuroblastoma cell lines or primary rodent neurons where GCase enzyme activity or *GBA1* expression levels are exogenously modulated by treatment with the GCase suicide inhibitor conduritol B epoxide (CBE) (Manning-Bog et al., 2009; Cleeter et al., 2013; Dermentzaki et al., 2013), transfection with *GBA1*-specific siRNAs (Mazzulli et al., 2011), or over-expression of plasmids containing mutant *GBA1* (Cullen et al., 2011). Although these models have proven useful, exogenous manipulation of GCase or *GBA1* expression often creates unwanted off-target effects. Primary neuronal cultures from one mouse model were used to probe mitochondrial function in GD (Osellame and Duchen, 2013; Osellame et al., 2013). Recently, the development of induced pluripotent stem cell (iPSC) lines from GD patients and carriers has gained popularity, providing the opportunity to develop cell cultures of previously inaccessible diseased human neurons (Tiscornia et al., 2013; Woodard et al., 2014; Sun et al., 2015; Schondorf et al., 2014; Awad et al., 2015). The main disadvantages of both primary rodent neuronal cultures and iPSC-generated neurons are low cell culture yield and the labor-intensiveness of establishment and maintenance. We hypothesized that immortalized GD neurons derived from a GD mouse model could provide a high-yield, easy-to-maintain alternative for investigations of the cellular mechanisms involved in GD. Such immortalized neurons could also have utility for the evaluation of novel therapeutics and the validation of different reagents and antibodies.

Immortalization of primary cells is accomplished by exogenous introduction of immortalizing genes such as the SV40 large T antigen (SV40-T), which increases lifespan and induces unlimited proliferation by inactivation of the cell-cycle suppressors pRb, SEN6 and p53 (Ozer et al., 1996; Tevethia et al., 1998; Manfredi and Prives, 1994; Ozer, 2000; Jha et al., 1998). Neurons are terminally differentiated post-mitotic cells, which makes gene delivery via traditional transfection methods difficult. Lentiviral expression vectors have the ability to transduce proliferating and non-proliferating cells, and have been used for infection of primary rodent neuronal cultures (Lewis et al., 1992; Weinberg et al., 1991; Zhang et al., 2006; Ding and Kilpatrick, 2013; Eleftheriadou et al., 2014; Li et al., 2012). In this study, we report the successful SV40-T-mediated immortalization of mouse cortical neurons derived from a previously established mouse model deficient in murine glucocerebrosidase (Tybulewicz et al., 1992).

## RESULTS

### The EF1α promotor drives expression in cultured mouse cortical cells

**Fig. 1.**
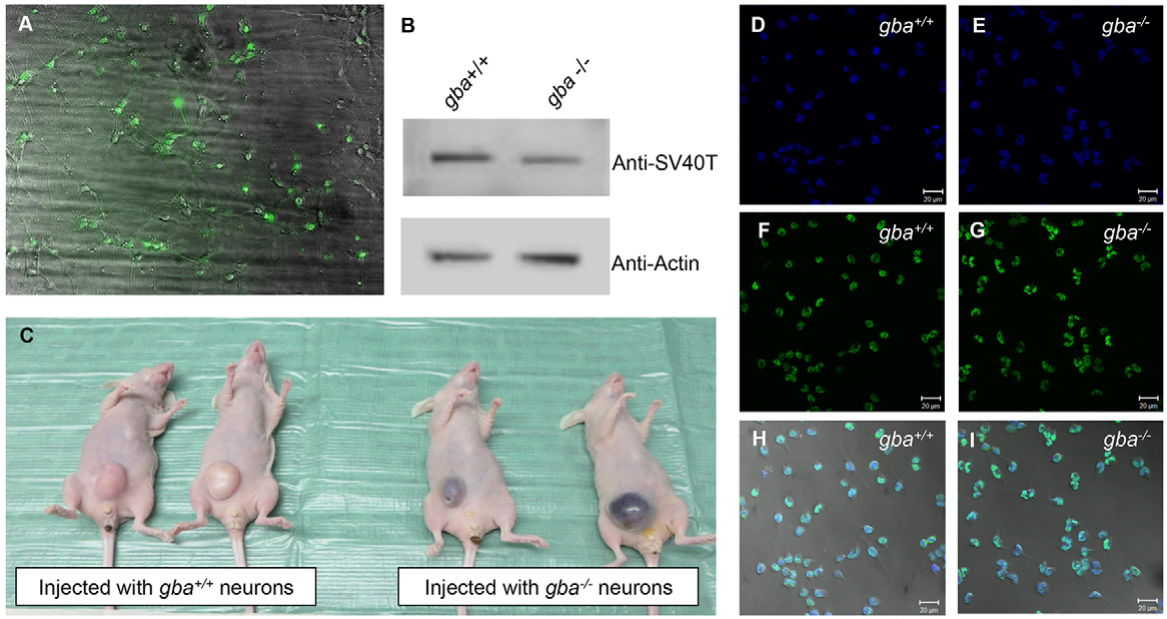
**Expression analysis and *in vivo* tumor formation of SV40-T immortalized mouse cortical cells.** (A) EGFP expression in primary C57BL/6 mouse cortical cell cultures five days after infection with EF1α-eGFP lentivirus at MOI 40. (B) Western blot analysis of protein lysates of *gba^+/+^* (lane 1) and *gba^−/−^* (lane 2) immortalized cortical cells with antibodies against SV40-T and β-actin (protein loading control). SV40-T protein expression was detected in both *gba^+/+^* and *gba^−/−^* immortalized cells. (C) *In vivo* intraperitoneal tumor formation after injection of 10^6^ cells of *gba^+/+^* and *gba^−/−^* immortalized cells in five Swiss nu/nu mice per genotype. Four weeks after injection, mice injected with *gba^+/+^* cells developed visible solid tumors (left panel) whereas mice injected with *gba^−/−^* cells developed solid bloody tumors (right panel). Immortalized *gba^+/+^* and *gba^−/−^* mouse cortical cells were stained for DAPI (D,E), anti-SV40-T (F,G), and the image merged with bright-field for visualization of neuron morphology (H,I). DAPI and SV40-T co-localize in the nucleus (H,I). Scale bar: 20 μm.

Several independent studies established that promotor determination for optimal gene expression in a specific cell type is beneficial (Day et al., 2009; Tsuchiya et al., 2002). Therefore, we tested a panel of eight different promoters fused to enhanced green fluorescent protein (eGFP) for their expression capacity in C57BL/6 primary mouse neuronal cultures (Table 1). Brains from 17E C57BL/6 embryos were harvested and neuronal cultures were established. Six-day-old primary embryonic cortical neuronal cultures were transduced with lentivirus containing recombinant genes encoding mPol2, Grp78, FerH, CAG, CMV13, PGK, EF1α or TRE-Tight fused to eGFP. We identified the EF1α promotor as an optimal promoter for primary mouse cortical cells with robust eGFP expression five days after infection at multiplicity of infection (MOI) 40 (Fig. 1A). At this point, the infected primary mouse cortical cell cultures consist of a mixed population of neurons and glial cells. Our findings were in agreement with previous studies where EF1α was identified as a suitable promoter for expression in rat cortical cell cultures and mouse neural precursor cells (Tsuchiya et al., 2002; Zeng et al., 2003).

**Table 1.**
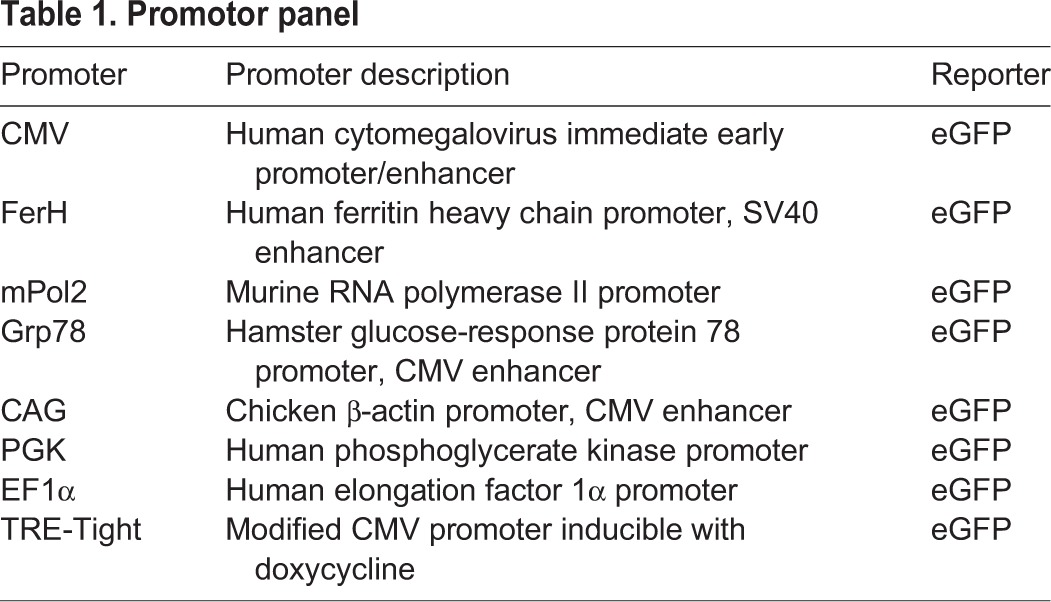
**Promotor panel**

### EF1α-driven expression of SV40-T immortalizes primary cortical mouse cells

Exogenous introduction of the immortalizing gene *SV40-T* is known to induce proliferation and increase life-span by inactivation of key cell-cycle suppressors (Ozer et al., 1996; Tevethia et al., 1998; Manfredi and Prives, 1994; Ozer, 2000; Jha et al., 1998).

Cultures of primary embryonic cortical cells were established from a previously described mouse model representative of type 2 GD (Tybulewicz et al., 1992). Six-day-old cultures of *gba^−/−^* and *gba^+/+^* cortical cells were infected with EF1α-SV40-T lentivirus at MOI 40. Five days after infection, SV40-T expressing cells were selected by treating with puromycin for four weeks. After selection, the immortalized cortical cell cultures were passaged and expression of SV40-T was confirmed in protein lysates of immortalized cultures of each genotype by western blotting (Fig. 1B). Laser scanning confocal microscopy on cells stained with a nuclear DAPI stain (Fig. 1D,E) and an anti-SV40T antibody (Fig. 1F,G) revealed localization of SV40-T in the nucleus (Fig. 1H,I) in immortalized neurons of both genotypes. To investigate whether SV40-T expression induces *in vivo* tumor growth, we performed intraperitoneal injection with 10^6^ cells of each genotype in Swiss nu/nu mice and monitored tumor formation. All mice displayed visible tumors four weeks after injection (Fig. 1C).

### Establishment of CD24-positive immortalized neurons

**Fig. 2.**
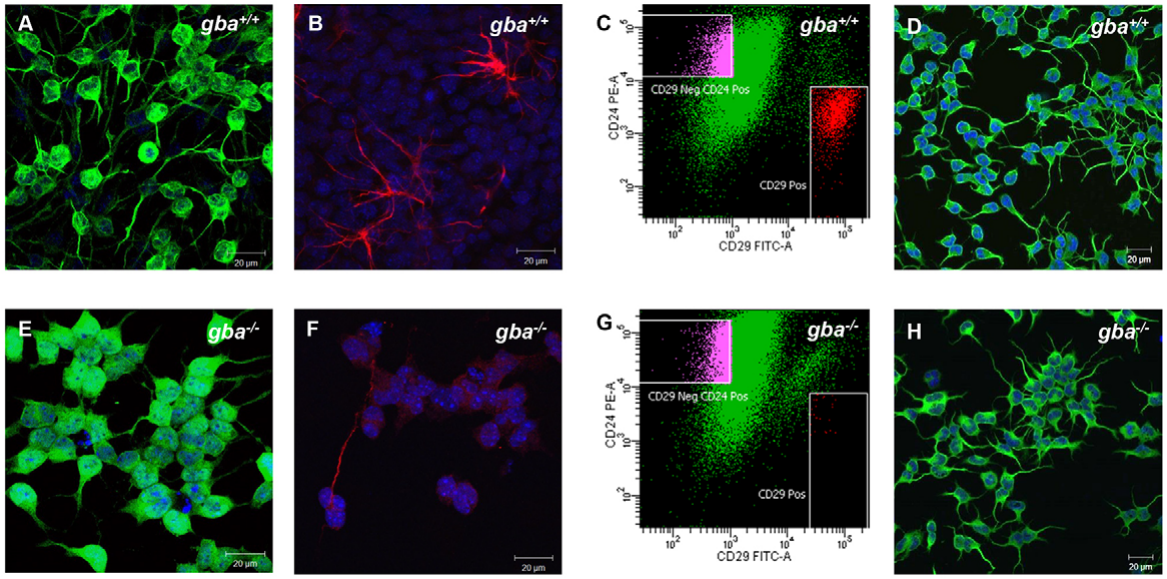
**Establishment of CD24-positive *gba^+/+^* and *gba^−/−^* neuron cultures.** Expression of (A,E) MAP-2 and (B,F) GFAP in SV40-T immortalized *gba^+/+^* and *gba^−/−^* cells before FACS. Both MAP-2 and GFAP are expressed in *gba^+/+^* cultures. GFAP is only sporadically expressed in *gba^−/−^* cultures. (C,G) FACS of SV40-T immortalized *gba^+/+^* and *gba^−/−^* cells with antibodies against CD24 and CD29 surface markers. (D,H) Expression of MAP-2 and GFAP in SV40-T immortalized *gba^+/+^* and *gba^−/−^* neuronal cultures after FACS. No GFAP-positive cells are detected. Scale bar: 20 μm.

**Fig. 3.**
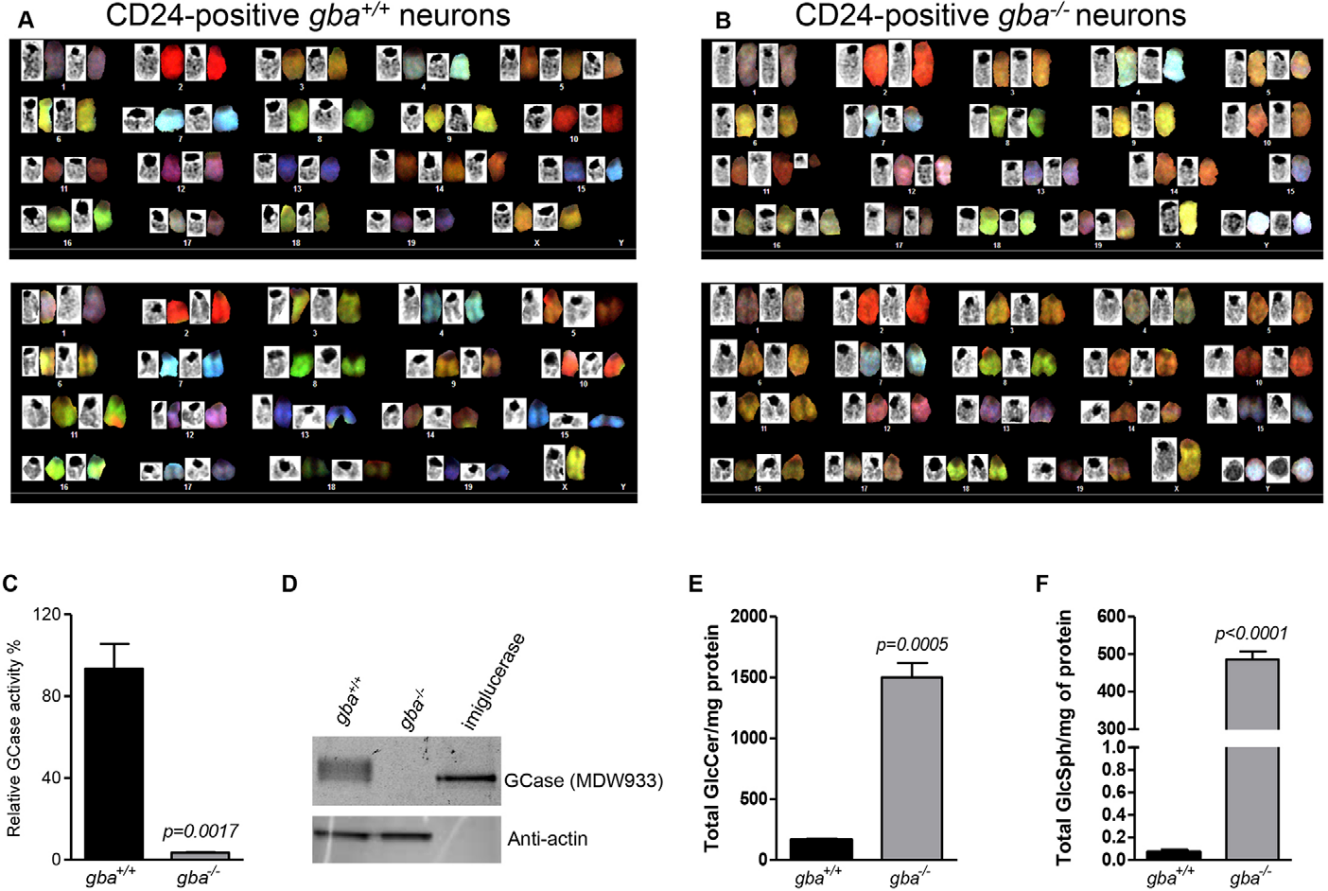
**Karyotyping, GCase enzyme activity, protein expression and substrate levels in immortalized *gba^+/+^* and *gba^−/−^* neurons.** (A,B) Karyotyping of immortalized CD24-positive *gba* neurons. Two examples each of heterogeneous karyotypes observed for immortalized *gba^+/+^* and *gba^−/−^* mouse neurons. (C) GCase enzyme activity in SV40-T immortalized *gba^+/+^* and *gba^−/−^* mouse neurons. Relative percentage GCase enzyme activity of g*ba^−/−^* (3.40±0.36%) immortalized neurons is significantly lower compared with *gba^+/+^* immortalized neurons (93.44±12.26%) (*P*=0.0017). Each enzyme assay was performed three independent times (*n*=3) in triplicates. The data are represented as relative mean values±s.e.m. (D) 15 µg of neuronal protein lysate was incubated with 100 nM of the GCase-specific MDW933 fluorescent inhibody. GCase protein expression is absent in *gba^−/−^* immortalized neurons (lane 2). Recombinant imiglucerase was loaded as a positive control (lane 3). (E) *Gba^−/−^* immortalized neurons show significantly increased GlcCer storage compared with *gba^+/+^* immortalized neurons (1500±118.9 vs 165.1±6.14 GlcCer/mg protein; *P*=0.0005). (F) *Gba^−/−^* immortalized neurons show significantly increased GlcSph storage compared with *gba^+/+^* immortalized neurons (485.7±21.24 vs 0.071±0.02 GlcSph/mg protein; *P*<0.0001). Each substrate storage assay was done four independent times (*n*=4). The data are represented as mean values±s.e.m.

As previously mentioned, cultures established from mouse cortex contain a mixed cell population consisting of neurons and glial cells. We performed immuno-cytochemistry on the established SV40-T immortalized *gba^+/+^* and *gba^−/−^* cultures with the neuronal marker microtubule-associated protein-2 (MAP-2) and the astroglial marker glial fibrillary acidic protein (GFAP). The immortalized cultures of each genotype were positive for both microtubule-associated protein-2 (MAP-2) and glial fibrillary acidic protein (GFAP) (Fig. 2A,B,E,F). Interestingly, GFAP-positive cells were only sporadically detected in *gba^−/−^* cultures (Fig. 2F). We next selected cells that were negative for the neural stem and precursor cell marker CD29 and positive for the differentiated neuron marker CD24 (Pruszak et al., 2007). Immortalized cortical cell cultures of both genotypes were subjected to positive CD24 selection and negative CD29 selection using fluorescence-activated cell sorting (FACS) (Fig. 2C,G). After FACS, the vast majority of cells stained positive for MAP-2, whereas GFAP-positive cells were absent (Fig. 2D,H). Multiple studies have shown that SV40-T immortalization of cells induces aberrant karyotypes (Bloomfield and Duesberg, 2015; Stoner et al., 1991; Toouli et al., 2002), a phenomenon also frequently observed in widely used cell lines such as HeLa and HEK293 (Landry et al., 2013; Stepanenko and Dmitrenko, 2015). Chromosome analysis on the *gba^+/+^* and *gba^−/−^* CD24-positive immortalized cortical neurons revealed aberrant heterogeneous karyotypes, which was expected as the cultures are heterogeneous and not clonal. Chromosomal abnormalities include numerical abnormalities (+Y, +5, +14, +16) and morphological abnormalities like translocation 5;19 (Fig. 3A,B).

### *gba^−/−^* immortalized neurons have deficient GCase enzymatic activity and show substrate storage

The previously described neonatal-lethal mouse model of type 2 GD is characterized by severe GCase enzyme deficiency and accumulation of GlcCer in the brain (Tybulewicz et al., 1992). We analyzed the CD24-positive immortalized mouse neurons and found that these characteristics were preserved. *gba^−/−^* immortalized neurons showed a severe deficiency in enzyme activity (3.40±0.36% relative GCase activity, mean±s.e.m.) compared with *gba^+/+^* immortalized neurons (93.44±12.26% relative GCase activity), which was highly significant (*P*=0.0017) (Fig. 3C). GCase protein could not be detected in *gba^−/−^* lysates with the previously described GCase-specific inhibody MDW933 (Witte et al., 2010) (Fig. 3D, lane 2). Subsequently, we performed LC-MS-MS analysis of glycosphingolipids, which revealed significant GlcCer storage (*P*=0.0005) in *gba^−/−^* immortalized neurons (1500±118.9 GlcCer/mg protein) compared with *gba^+/+^* immortalized neurons (165.1±6.14 GlcCer/mg protein) (Fig. 3E). Significant GlcSph storage (*P*<0.0001) was observed in *gba^−/−^* immortalized neurons (485.7±21.24 GlcSph/mg protein) compared with *gba^+/+^* immortalized neurons (0.071±0.02 GlcSph/mg protein) (Fig. 3F). Next, we analyzed expression of alpha-synuclein (α-syn), which is a small protein (14 kDa) with abundant expression in neurons. Aggregation of α-syn results in the formation of insoluble oligomeric and fibrillar inclusions, a prominent pathological feature in the brain of individuals with synucleinopathies (Fearnley and Lees, 1991; Puschmann et al., 2012; Puschmann, 2013). High α-syn protein expression has been reported in cultured primary mouse neurons, including *gba*-deficient mouse neurons (Osellame et al., 2013). Western blot analysis with two different antibodies against α-syn did not show any monomeric α-syn protein expression in *gba^+/+^* and *gba^−/−^* immortalized mouse neurons (data not shown). Although it has been shown that treatment with retinoic acid, an agent that induces neuronal differentiation (Manning-Bog et al., 2009), and valproic acid, a histone deacetylase inhibitor, can increase α-syn protein and mRNA levels in primary cultures of rat cerebellar granule cells (Leng and Chuang, 2006), treating immortalized *gba^+/+^* and *gba^−/−^* cortical neuron cultures with 1 mM valproic acid for 4 days or 10 μM retinoic acid for 6 days did not result in endogenous α-syn protein expression (data not shown).

### *gba^−/−^* immortalized neurons have enlarged lysosomes

**Fig. 4.**
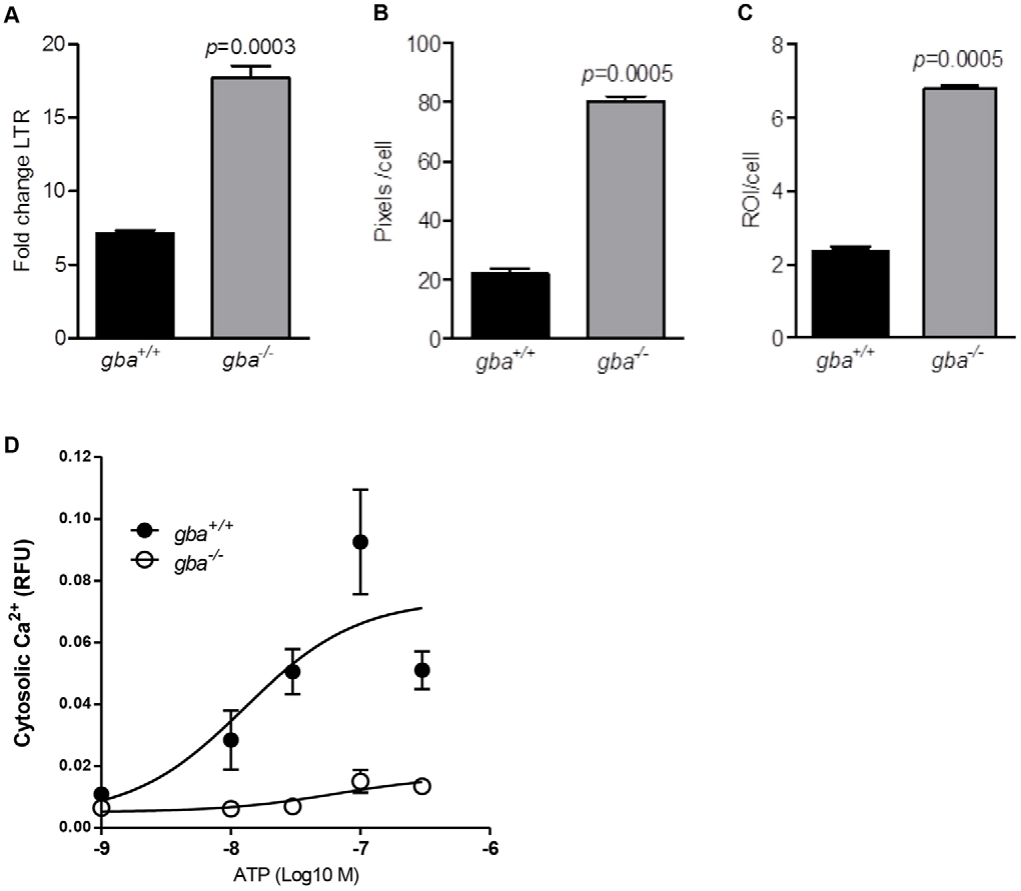
**Quantitative analysis of lysosomes and ATP-dependent Ca^2+^ influx in immortalized *gba ^+/+^* and *gba^−/−^* neurons.** (A) FACS analysis of LysoTracker stained *gba^−/−^* neurons and *gba^+/+^* (fold-change 17.7±0.83 vs 7.13±0.23). Data from three independent experiments (*n*=3) showed significantly increased LysoTracker staining in *gba^−/−^* neurons (*P*=0.0003). (B) Total LysoTracker^®^ pixels per cell was significantly higher in *gba^−/−^* cells compared with *gba^+/+^* cells (80.22±1.79 vs 22.12±1.85; *P*=0.0005). (C) The number of ROIs acquired per cell was significantly higher in *gba^−/−^* cells compared with *gba^+/+^* cells (6.79±0.11 vs 2.36±0.13; *P=*0.0005). (D) After treatment of cells with different concentrations of ATP (3 µM, 1 µM, 0.3 µM, 0.1 µM), Ca^2+^ influx in *gba^+/+^* (black dots) was significantly higher (*P*=0.0036) compared with *gba^−/−^* (white dots). Experimental data was normalized to numbers of cells with the CellTag 700 assay. Each experiment included quadruplicate samples and was repeated two times (*n*=2). The data are represented as mean values±s.e.m.

Gaucher cells, engorged macrophages with lysosomal GlcCer accumulation, are primarily found in the spleen, liver and bone marrow of individuals with GD (Sidransky, 2012). These typical enlarged, lipid-laden lysosomes were previously identified in the liver and bone marrow of *gba^−/−^* mice (Tybulewicz et al., 1992). We investigated whether the CD24-positive *gba*^−/−^ neurons, which show GlcCer and GlcSph substrate storage (Fig. 3E,F), have enlarged lysosomes by estimating the lysosomal volume per cell in *gba^+/+^* and *gba^−/−^* neurons using LysoTracker^®^, which specifically stains acidic endosomal compartments. Using an established FACS analysis method to calculate the fold-change in LysoTracker^®^ as the ratio of the geometric means of LysoTracker^®^ in stained and unstained samples (Rodriguez-Gil et al., 2013), we detected a significant increase (*P*=0.0003) in intensity of LysoTracker^®^ staining of *gba^−/−^* neurons (fold-change 17.7±0.83) compared with *gba^+/+^* (fold-change 7.13±0.23) (Fig. 4A). This was confirmed with a 96-well high-throughput imaging assay (Acosta et al., 2015) where enlarged lysosomes had increased LysoTracker^®^ signal intensity, reflecting the size and number of acidic endosomes. After segmentation, the number of LysoTracker^®^ objects or region of interest (ROI) and total pixels per image were quantified and divided by the number of cells in the image. Lysosomal volume in *gba^−/−^* cells (80.22±1.79), measured as total LysoTracker^®^ pixels per cell, was almost four times greater than *gba^+/+^* cells (22.12±1.85) (Fig. 4B). The number of ROIs acquired per cell was also significantly higher in *gba^−/−^* (6.79±0.11) compared with *gba^+/+^* (2.36±0.13) (Fig. 4C).

### *gba*^−/−^ neurons show an impaired ATP-dependent Ca^2+^ response

Previous studies have shown that rodent neuronal cell models and brain samples from individuals with GD showed increased Ca^2+^ release from intracellular Ca^2+^ stores such as the endoplasmic reticulum (ER) into the cytosol (Korkotian et al., 1999; Pelled et al., 2000, 2005; Lloyd-Evans et al., 2003a,b). Cytosolic Ca^2+^ can also be modulated by influx of Ca^2+^ from the extracellular environment into the cytosol via ATP-selective purinergic receptors (P2X receptors), which are expressed on the plasma membrane of glial cells and neurons (Saez-Orellana et al., 2015). We measured ATP-dependent Ca^2+^ influx with the intracellular Ca^2+^ marker Fluo-4 AM (Fig. 4D). Immediately after treatment with 3 µM, 1 µM, 0.3 µM or 0.1 µM of ATP, we observed a significant dose-dependent elevation of Ca^2+^ influx in *gba^+/+^* (black dots) compared with *gba^−/−^* neurons (white dots) (*P*=0.0036), which suggest that treatment with ATP failed to induce Ca^2+^ influx in *gba^−/−^* neurons (Fig. 4D). The Ca^2+^ influx data was normalized to the number of cells seeded in each well of the 96-well plate with the CellTag 700 assay.

## DISCUSSION

The establishment of high-yield and easy-to-manipulate immortalized *gba^−/−^* neuronal cells from a previously described *gba-*deficient mouse model can serve as a valuable tool to test newly discovered cellular mechanisms or therapeutics. This is particularly useful before moving to primary neurons or iPSC-derived neurons, which have a low cell culture yield, are costly, and are labor-intensive to establish and maintain. Most other available neuronal cell models rely on the GCase suicide inhibitor CBE, *gba1* gene silencing, or exogenous introduction of *GBA1* mutants to modulate GCase (Manning-Bog et al., 2009; Cleeter et al., 2013; Dermentzaki et al., 2013; Mazzulli et al., 2011; Cullen et al., 2011).

Primary neurons are post-mitotic cells that are terminally differentiated. However, upon introduction of SV40-T, primary cells are forced into proliferation resulting in increases in life-span (Ozer et al., 1996; Tevethia et al., 1998; Manfredi and Prives, 1994; Ozer, 2000; Jha et al., 1998). After SV40-T-mediated immortalization, we obtained actively dividing cortical cells that retained the neuronal marker MAP-2 and glial marker GFAP. FACS on cortical cell cultures allowed us to isolate CD24-positive neurons, which were exclusively positive for MAP-2 and negative GFAP. Cellular defects observed in the mouse model such as the absence of GCase protein expression and enzyme activity as well as elevated levels of GlcCer and GlcSph substrate (Tybulewicz et al., 1992) were all maintained in our established SV40-T immortalized *gba^−/−^* neurons. Previously, lysosomes in fibroblasts of the rare recessive lysosomal storage disorder Niemann–Pick C disease (NPC1; MIM #257220) were analyzed with FACS using LysoTracker^®^ staining, a fluorophore selective for acidic cellular organelles. The LysoTracker^®^ signal was significantly elevated in NPC1 patient fibroblasts and these lysosomal alterations seemed to directly correlate to the time of onset of neurological disease symptoms (Rodriguez-Gil et al., 2013). Using the same method, we found a significant increase in LysoTracker^®^ intensity in *gba^−/−^* immortalized neurons; this was confirmed by an established high content fluorescence microscopy assay (Acosta et al., 2015). This finding indicates that substrate storage in *gba^−/−^* neurons could lead to enlarged lysosomes.

Mutations in *GBA1* are an important risk factor for the development of several synucleinopathies including PD, DLB and MSA (Mitsui et al., 2015; Nalls et al., 2013; Sidransky et al., 2009). The main pathological feature is the presence of α-syn aggregates and inclusions (Fearnley and Lees, 1991; Puschmann et al., 2012; Puschmann, 2013). Recent *in vitro* and *in vivo* studies favor a reciprocal relationship between GCase and α-syn, where diminished GCase enzyme activity or protein expression increases accumulation of α-syn or where increased α-syn protein levels result in reduced GCase activity and protein expression (Gegg et al., 2012; Murphy and Halliday, 2014; Chiasserini et al., 2015; Mazzulli et al., 2011; Sardi et al., 2015). Additional evidence in favor of this model came from cultures of primary neurons from the midbrain of an inducible neuronopathic GD type 2 mouse model (*gba^−/−^*) with significant accumulation of endogenous α-syn (Osellame et al., 2013), suggesting that absent GCase favors α-syn accumulation. We analyzed expression of monomeric α-syn in our SV40-T immortalized *gba^−/−^* and *gba^+/+^* neurons but failed to detect α-syn protein, which is similar to what has previously been described in actively dividing neuroblastoma cell lines such as SH-SY5Y. In contrast to other published rodent and human neuronal lines, we did not observe enhanced α-syn protein levels when immortalized neurons were treated with retinoic acid or the histone deacetylase inhibitor valproic acid (Manning-Bog et al., 2009; Leng and Chuang, 2006). Because the immortalized CD-24-positive mouse neurons do not express endogenous α-syn, caution has to be taken when using these cells as a tool to investigate the relationship between GCase and α-syn. To address this, introduction of exogenous α-syn by plasmid transfection or lentiviral infection could be performed, which is a strategy used for actively dividing neuroblastoma cells that lack endogenous α-syn such as SH-SY5Y (McNeill et al., 2014; Dermentzaki et al., 2013).

Previous studies showed increased Ca^2+^ release into the cytosol from intracellular Ca^2+^ stores in cells and tissues with GlcCer and GlcSph storage. Current literature proposes that elevated GlcCer levels contribute to neuronal cell death via increased ryanodine receptor-dependent Ca^2+^ release, whereas GlcSph storage enhanced Ca^2+^ release via the inositol (1,4,5)-trisphosphate receptor (InsP3R) and sarcoplasmic/endoplasmic reticulum Ca^2+^-ATPase (SERCA) (Korkotian et al., 1999; Pelled et al., 2000, 2005; Lloyd-Evans et al., 2003a,b). Cytosolic Ca^2+^ concentration also depends on the influx of Ca^2+^ from the extracellular environment, which is dependent upon ATP-selective P2X receptors expressed on the plasma membrane of glial cells and neurons (Saez-Orellana et al., 2015; Volonte et al., 2003). ATP-dependent P2X receptor-mediated Ca^2+^ influx in neurons is described to be involved in the modulation of neurotransmitter release, synaptic plasticity (Saez-Orellana et al., 2015), and even lysosomal dysfunction and α-syn aggregation (Gan et al., 2015). Further research is needed to uncover the exact role of the different P2X receptors in the impaired ATP-dependent Ca^2+^ influx in *gba^−/−^* neurons and the associated implications on neurological function.

Multiple studies have shown that SV40-T-mediated immortalization of primary cells induces aberrant karyotypes (Bloomfield and Duesberg, 2015; Stoner et al., 1991; Toouli et al., 2002), which is also frequently observed in widely used cell lines such as HeLa and HEK293 (Landry et al., 2013; Stepanenko and Dmitrenko, 2015). Chromosomal analysis of the immortalized *gba^+/+^* and *gba^−/−^* CD24-positive neuronal cultures revealed heterogeneous aberrant karyotypes, which was expected as the cultures are not clonal. We are confident that the observed phenotypes are related to absence of *gba1* and not random integration location. Recently, Gramlich and co-workers used this immortalized *gba^−/−^* cell model as an *in vitro* model for the evaluation of neuronal uptake and substrate turnover of newly developed recombinant GCase proteins. Uptake of GCase proteins into *gba^−/−^* neurons showed a significant reduction of GlcSph storage (Gramlich et al., 2016). We were aware that phenotype drifting might happen; however, confirmation of experimental reproducibility on different cell passage numbers revealed little variation.

In summary, we describe the first successful SV40-T-mediated immortalization of mouse cortical neurons derived from a previously established *gba*-deficient mouse model (Tybulewicz et al., 1992). The immortalized *gba^−/−^* neurons express neuronal markers and exhibit a *gba*-deficient phenotype characterized by GCase enzyme deficiency, absent GCase protein expression, and significant accumulation of GlcCer and GlcSph. This original neuronal cell line constitutes a relevant, high-yield, and easy-to-manipulate *in vitro* tool for not only assessing the molecular and cellular defects associated with GD, but also for developing and evaluating novel therapeutic strategies (Gramlich et al., 2016).

## MATERIALS AND METHODS

### Culturing of primary mouse neurons

Animal studies were in accordance with protocol G-05-4, which was approved by NHGRI Animal Care and Use Committee (National Institutes of Health, Bethesda, MD, USA). Brains from *gba-*deficient or C57BL/6 mouse embryos at day 17 (17E) were harvested and placed in cold dissecting media [10 ml L15 media, 0.15 ml HEPES buffer, and 0.15 ml pen/strep (Life Technologies, Grand Island, NY, USA)]. The meninges was removed, cortices were isolated, minced and incubated for 45 min at 37°C in papain solution with DNaseI (Worthington Biochemical Corporation, Lakewood, NJ, USA). Minced cortices were gently triturated (10-15 times) followed by centrifugation at 164 ***g*** for 5 min at room temperature (RT). The supernatant was removed and cell pellet was resuspended in 500 µl papain inhibitor (Worthington Biochemical Corporation) with DNaseI. After 10 min, stop solution was aspirated and 10 ml of a 10/10 solution HBSS (Life Technologies), 0.1 g trypsin inhibitor (Sigma Aldrich, St. Louis, MO, USA), 0.1 g BSA fraction V (Sigma Aldrich) was added to the settled cells. The cells were spun down at 105 ***g*** for 10 min. After removing the supernatant, cell pellets were resuspended in 2 ml neurobasal growth media [500 ml neurobasal media, 1:50 (v/v) B27, 1:200 (v/v) glutamine, and 25 mM HEPES (Life Technologies)]. 500 µl of cell suspension with 2.5 ml of neurobasal growth media was added to each well of a 6-well plate pre-coated with poly-L-lysine (Sigma Aldrich). 50% of the neurobasal growth media was changed every 3 days.

### Chemicals and antibodies

Conduritol B epoxide (CBE) (Sigma Aldrich), an irreversible inhibitor of glucocerebrosidase, was dissolved in DMSO to a stock solution of 100 μM. Valproic acid (Sigma Aldrich), a histone deacetylase inhibitor, was dissolved in ethanol to a stock solution of 200 mM. The following antibodies were used for western blotting: anti-SV40T mouse monoclonal (Santa Cruz Biotechnology Inc., CA, USA, sc-148, 1:2000), anti-α synuclein rabbit polyclonal (Santa Cruz Biotechnology Inc., sc-7011-R, 1:1000), anti-α synuclein polyclonal (EMD Millipore, Billerica, MA, USA, AB5038, 1:1000), anti-beta Actin conjugated to HRP (Abcam, Cambridge, MA, USA, ab20272, 1:5000), and MAP-2 rabbit polyclonal (Cell Signaling, Danvers, MA, USA, 8707S, 1:1000). The following antibodies were used for immunocytochemistry: anti-SV40T mouse monoclonal (Santa Cruz Biotechnology Inc., sc-148, 1:100), anti-GFAP rabbit polyclonal (Abcam, ab16997, 1:100), anti-MAP2 chicken polyclonal (Abcam, ab92434, 1:100).

### Lentiviral vector production and transfection

Lentiviral plasmid DNA was co-transfected with ViraPower Lentiviral packaging plasmids (Life Technologies) into HEK293T cells to generate VSV-g pseudotyped lentivirus particles. The cells received fresh DMEM media (Life Technologies) 24 h post-transfection and the culture supernatant was harvested 48 h post-transfection. The crude lentivirus stock was filtered, centrifuged and concentrated using Amicon Ultra-15 columns (EMD Millipore). Virus titer was determined with the Global Ultra Rapid Lentiviral Titer kit (System Biosciences, Mountain View, CA, USA) according to the manufacturer's guidelines. Lentiviral particles were infected at multiplicity of infection (MOI) 40 with the TransDux reagent according to the manufacturer's guidelines (System Biosciences).

### Promotor testing and immortalization of primary mouse neurons

Packed lentiviral particles, containing enhanced green fluorescent protein (eGFP) driven by distinct promoters (mPol2, Grp78, FerH, CAG, CMV13, PGK, EF1α, TRE-Tight), were ordered from Leidos Biomedical Research Inc. (Frederick, MD, USA). 6-day-old primary cortical neuron cultures from C57BL/6 mice were infected at MOI 10, 20 and 40 with the TransDux reagent. eGFP expression was evaluated after 5 days of infection with a fluorescence microscope (Zeiss, San Diego, CA, USA). Packed lentiviral particles containing EF1α-SV40T were ordered from Leidos Biomedical Research Inc and 6-day-old primary cortical neuron cultures from *gba^−/−^* and *gba^+/+^* mice were infected at MOI 40 with TransDux reagent (System Biosciences). After 4 days of infection, the cultures were treated with 1 µg/ml of puromycin (Sigma Aldrich) for 4 weeks; the media was changed every 3 days.

### *In vivo* tumor formation

Animal studies were in accordance with a protocol approved by the Local Ethics Committee of Ghent University Hospital (Ghent, Belgium). At the age of 5 weeks, female Swiss nu/nu mice (five mice per genotype; Charles River Laboratories, Brussels, Belgium) were injected intraperitoneally with 10^6^ cells resuspended in 100 μl matrigel. Tumor growth was assessed after 4 weeks of injection.

### Spectral karyotyping

Metaphase slides were prepared after mitotic arrest with 2-4 h Colcemid (0.015 μg/ml; Thermo Fisher Scientific), 20 min hypotonic treatment (0.075 mol/l KCl, 37°C), and fixation with methanol–acetic acid (3:1). For spectral karyotyping we used commercial SKY probe (Applied Spectral Imaging Inc., Carlsbad, CA, USA) allowing the visualization of the individually colored chromosomes. This technique is used to identify structural and numerical chromosome aberrations in mouse cell lines (Schrock et al., 1996).

### Immunocytochemistry and laser scanning confocal microscopy

Cells were grown for 48 h on Lab-Tek chamber slides (Thermo Scientific, Waltham, MA, USA). Cells were fixed in 4% paraformaldehyde (Electron Microscopy Sciences, Hatfield, PA, USA), blocked in 1× PBS (Life Technologies) containing 0.1% saponin (Sigma Aldrich), 100 μM glycine (Sigma Aldrich), and 2% donkey serum (Jackson Immunoresearch Laboratories Inc, West Grove, PA, USA) followed by a 4°C overnight incubation with primary antibodies. Cells were washed with 1× PBS and incubated with the following secondary antibodies (all from Life Technologies): anti-mouse Alexa Fluor 488 (A-11001; 1:300), anti-chicken Alexa Fluor 488 (A-11039; 1:300), anti-rabbit Alexa Fluor 555 (A-21428; 1:300), for 1 h at RT, washed again, and mounted with Prolong Gold antifade reagent with or without DAPI (Life Technologies). Cells were imaged with a Zeiss 510 META confocal laser-scanning microscope (Carl Zeiss Microscopy, Munich, Germany) using a 488 argon, a 543 HeNe, and a diode laser. Images were acquired using a Plan NeoFluar 40×/1.3 oil DIC objective or a Plan Apochromat 63×/1.4 oil DIC objective. Bright-field images were obtained for visualization of neuron morphology.

### Fluorescence-activated cell sorting (FACS)

Neurons and glial cells of immortalized *gba^−/−^* and *gba^+/+^* neuronal cultures were separated by FACS. Cells of each genotype were labeled with FITC hamster anti-rat CD29 and PE rat anti-mouse CD24 (BD Biosciences, San Jose, CA, USA). Single-stained and unstained cells were used as a control. Cells were sorted using a BD FACSAriaII cytometer (BD Biosciences). Results were analyzed with FACSDiva software version 6.1.3 (BD Biosciences).

### Western blotting

The amount of protein for each sample was determined by DC™ protein assay (Bio-Rad Laboratories, Hercules, CA, USA). An equal amount of protein for each sample was loaded onto a 4-20% Mini-PROTEAN^®^ TGX™ gel (Bio-Rad Laboratories). After blotting with the Trans-Blot Turbo transfer system (Bio-Rad Laboratories), PVDF membranes (Bio-Rad Laboratories) were blocked for 1 h at RT in blocking solution [1× PBS, 0.5% (v/w) milk, 0.1% Tween (Sigma Aldrich)]. PVDF membranes were probed overnight with primary antibodies in blocking solution at 4°C. PVDF membranes were washed 3×5 min at RT with blocking solution. This was followed by incubation with HRP-coupled secondary anti-mouse or anti-rabbit antibodies (Amersham Biosciences, Piscataway, NJ, USA, NA931 and NA934, respectively, 1:4000). PVDF membranes were washed 3×5 min with blocking solution followed by 3×5 min with 1× PBS plus 0.1% Tween. The antigen-antibody complexes were detected with an Enhanced Chemiluminescence (ECL) kit (Amersham Biosciences).

### Quantification of GCase protein levels

Florescent activity-based probes specific for GCase (MDW933) were synthesized at the Imaging Probe Development Center (National Heart Lung and Blood Institute, Bethesda, MD, USA) as previously described (Witte et al., 2010). Total protein concentration of each sample was measured using a Bradford assay according to the manufacturer's guidelines (Bio-Rad Laboratories). Cell homogenate was incubated with 1 μM of green fluorescent MDW933 probe in citrate phosphate buffer (pH 5.4) at 37°C for 90 min. Samples were analyzed on a 4-20% Mini-PROTEAN^®^ TGX™ gel (Bio-Rad Laboratories) using 1.2 μM imiglucerase (Genzyme, Cambridge, MA, USA) with 1 μM MDW933 probe as a control. A Typhoon Variable Mode Imager (Amersham Biosciences, Piscataway, NJ, USA), set to excitation wavelength (λ_ex_)=488 nm and emission wavelength (λ_em_)=520 nm, was used to measure the fluorescent signal in the gel.

### GCase enzyme activity assay

Cell lysates were prepared in citrate-phosphate extraction buffer [150 mM citrate-phosphate buffer pH 5.4, 0.25% Triton X-100, protease inhibitor mix (Roche Diagnostics, Indianapolis, IN, USA)] and sonicated for 20 s at 50% amplitude using a mechanical tissue homogenizer (Omni International, Kennesaw, GA, USA). Cell homogenates were then centrifuged at 10,000 ***g*** for 15 min at 4°C. GCase enzyme activity was measured in an assay buffer composed of citrate-phosphate buffer (pH 5.4) with 10 mM 4-methylumbelliferyl-β-D-glucopyranoside (Sigma Aldrich). Cell homogenate (5 μl) was added to each individual well of a black 384-well plate (VWR International, Bridgeport, NJ, USA) and each sample was read in triplicate. To correct for cytosolic *gba2* activity, 5 μl of a 100 μM CBE solution (Sigma Aldrich) was added in triplicate and assay buffer was pipetted to each well to bring the total assay volume to 30 μl. The plate was briefly centrifuged and then incubated at 37°C for 1 h at 600 rpm. The reaction mixtures were quenched by the addition of 30 μl of stop solution (1 M glycine, pH 12.5) and fluorescence was measured using a FlexStation 3 Multi-Mode Microplate Reader (Molecular Devices, Sunnyvale, CA, USA), at λ_ex_=520 nm and λ_em_=440 nm. Enzyme activity was normalized based on total protein amount. Data were analyzed by a Student's two-tailed *t*-test and are represented as mean±s.e.m.

### FACS analysis for lysosomal size

*gba^−/−^* and *gba^+/+^* neurons were plated in 60 mm tissue culture dishes (VWR International) and grown to 80% confluency. The cells were given fresh neurobasal growth media 24 h before analysis. On the day of analysis, cells were incubated with neurobasal media supplemented with 1 μM Lysotracker Red DND-99 (Thermo Fisher Scientific) for 1 h at 37°C. FACS analysis was performed on a FACSCalibur (BD Biosciences, Franklin Lakes, NJ, USA) according to a previously described protocol (Rodriguez-Gil et al., 2013). Biological duplicates were analyzed in three independent experiments (*n*=3). Fold-change was calculated as the ratio of the geometric means of Lysotracker Red stained and unstained samples. Data were analyzed by a Student's two-tailed *t*-test and represented as mean±s.e.m.

### High-throughput imaging for lysosomal size

A recently described protocol was optimized for high-throughput imaging on SV40 immortalized *gba^−/−^* and *gba^+/+^* neurons (Acosta et al., 2015). The cell lines were plated in a black clear bottom 96-well plate in neurobasal media. Sixteen wells per cell line were plated. After 72 h, cells were treated with 1 µM Lysotracker Red (Life Technologies) for 20 min and washed with 1× PBS. Cells were fixed with 4% paraformaldehyde for 5 min followed by 3× washes with PBS. Cells were counterstained with 300 nM DAPI (Life Technologies). The BD Pathway^®^ 855 High Content Bioimager (BD Biosciences, Franklin Lakes, NJ) was used to acquire images in each well under identical settings for exposure, dynamic range, and laser autofocus. Images for each well were taken using a 2×2 montage with the 20× objective yielding an average of 400 cells per image (∼6400 cells per treatment). For each well, fluorescent signal (excitation/emission) was acquired at λ_ex_=560 nm and λ_em_=645 nm for the red signal and λ_ex_=380 nm and λ_em_=435 nm for the blue signal. Image segmentation was performed using Attovision^®^ software (BD Biosciences). Cell count was obtained using polygon segmentation for the nucleus DAPI signal. LysoTracker^®^ regions of interest (ROI) were obtained by polygon segmentation of one of each compartment detected in the red signal. Segmentation data was analyzed using BD Data Explorer^®^ software (BD Biosciences). An average of LysoTracker^®^ pixels per cell was calculated for each image. Averages of the sixteen images (*n*=16) were used to estimate the value of each treatment. Data were analyzed by a Student's two-tailed *t*-test and represented as mean±s.e.m.

### Lipidomics

Glycosphingolipids (glucosyl- and galactosyl-sphingosines, glucosyl- and galactosyl-ceramides) were extracted with methanol from mouse homogenized immortalized neurons containing N,N-dimethylgalactosylsphingosine and galactosylceramide (d18:1 8:0) as internal standards. Glycosphingolipid analysis was initially carried out for quantification of glycosylsphingosines and glucosylceramides using a Varian reverse phase C-18 metasil column that was connected to an API 4000 LC-MS-MS system (Applied Biosystems). Later isomer separation of glycosphingolipids was performed using a Supelco HILIC column for determination of the isomer composition. Positive ion electrospray method using MRM was used for both analyses. Data were normalized to protein content in the samples. Data was analyzed by a Student's two-tailed *t*-test and represented as mean±s.e.m.

### Calcium assay

On day one, SV40-T immortalized neurons were seeded at 70% confluency in a black flat clear bottom 96-well plate (Corning Inc, Kennebunk, ME). On day 3, neurobasal media was removed from the cells and assay buffer, which consists of physiological salt solution (PSS) buffer (126 mM NaCl, 5 mM KCl, 1.2 mM MgCl_2_, 10 nM HEPES, 10 nM glucose, 1 nM CaCl_2_)+0.2 mM sulfinpyrazone+0.1 mM CaCl_2_+12 µl pluronic F-127 (Life Technologies)+60 µg fluo-4 AM (Life Technologies), was added and incubated for 1 h at 37°C and 10% CO_2_. Assay buffer was removed and cells were washed once with 1× PBS followed by incubation with PSS buffer+0.2 mM sulfinpyrazone+0.1 mM CaCl_2_ for 10 min at 37°C and 10% CO_2_. The 96-well plate was inserted into the Flexstation 3 plate reader (Molecular Devices, Sunnyvale, CA) and the cells were treated with 10 µM, 3 µM, 1 µM, 0.3 µM and 0.1 µM of ATP dissolved in PSS buffer. PSS buffer without ATP was included as a negative control. The Flex mode setting was used with λ_ex_=494 nm and λ_em_=516 nm. ATP-dependent Ca^2+^ response was followed for 120 s over 10 s intervals with six readings per well. The experiment included quadruplicate samples per plate and was repeated two independent times. Dose-dependent curves were fitted and EC50 was calculated.

### CellTag 700 assay

After data acquisition on the Flexstation-3 plate reader, the cells in the black 96-well plate were fixed in 4% paraformaldehyde for 1 h at RT. This was followed by permeabilization with 0.1% Triton-X (Sigma Aldrich) for 10 min at RT. The cells were washed with 1× PBS followed by incubation with CellTag 700 (LI-COR Biosciences, Lincoln, NE, USA) (1:3000) for 1 h at RT. After washing twice with 1× PBS, the plate was dried and imaged on the LI-COR imaging station (LI-COR Biosciences).

### Statistical analysis

Data obtained from GCase enzyme activity, substrate storage, and lysosomes were analyzed by a Student's two-tailed *t*-test with GraphPad Prism^®^ software version 6.0 (GraphPad, San Diego, CA, USA) and data are represented as mean±s.e.m. For ATP-dependent Ca^2+^ response, dose curves were fitted and EC50 was calculated with the GraphPad Prism^®^ (version 6.0).
